# Relevance of Alternative Routes of Kynurenic Acid Production in the Brain

**DOI:** 10.1155/2018/5272741

**Published:** 2018-05-24

**Authors:** L. A. Ramos-Chávez, R. Lugo Huitrón, D. González Esquivel, B. Pineda, C. Ríos, D. Silva-Adaya, L. Sánchez-Chapul, G. Roldán-Roldán, V. Pérez de la Cruz

**Affiliations:** ^1^Laboratorio de Neurobiología de la Conducta, Departamento de Fisiología, Facultad de Medicina, Universidad Nacional Autónoma de México, Mexico; ^2^Departamento de Neuroquímica, SSA, Instituto Nacional de Neurología y Neurocirugía Manuel Velasco Suárez, Mexico City 14269, Mexico; ^3^Laboratorio de Neuroinmunología, SSA, Instituto Nacional de Neurología y Neurocirugía Manuel Velasco Suárez, Mexico City 14269, Mexico; ^4^Laboratorio Experimental de Enfermedades Neurodegenerativas, SSA, Instituto Nacional de Neurología y Neurocirugía Manuel Velasco Suárez, Mexico City 14269, Mexico; ^5^División de Neurociencias, SSA, Instituto Nacional de Rehabilitación, Mexico City 14389, Mexico

## Abstract

The catabolism of tryptophan has gained great importance in recent years due to the fact that the metabolites produced during this process, with neuroactive and redox properties, are involved in physiological and pathological events. One of these metabolites is kynurenic acid (KYNA), which is considered as a neuromodulator since it can interact with NMDA, nicotinic, and GPR35 receptors among others, modulating the release of neurotransmitters as glutamate, dopamine, and acetylcholine. Kynureninate production is attributed to kynurenine aminotransferases. However, in some physiological and pathological conditions, its high production cannot be explained just with kynurenine aminotransferases. This review focuses on the alternative mechanism whereby KYNA can be produced, either from D-amino acids or by means of other enzymes as D-amino acid oxidase or by the participation of free radicals. It is important to mention that an increase in KYNA levels in processes as brain development, aging, neurodegenerative diseases, and psychiatric disorders, which share common factors as oxidative stress, inflammation, immune response activation, and participation of gut microbiota that can also be related with the alternative routes of KYNA production, has been observed.

## 1. Kynurenic Acid (KYNA)

The main tryptophan (Trp) catabolism route is through the kynurenine pathway (KP), where the final product is the nicotinamide adenine nucleotide (NAD+) de novo production. NAD+ plays an essential role in metabolism and cellular energy homeostasis. NAD+/NADH ratio dysfunction is related to mitochondrial disorders, aging, and age-related diseases [1]. In humans, it is estimated that 95% of Trp is catabolized through KP [2]. Along with this pathway, some neuroactive metabolites are produced. One of them is kynurenic acid (KYNA), which is considered a natural antagonist for the glycine-B coagonist site of N-methyl-D-aspartate receptor (NMDAr). However, high micromolar concentrations of KYNA are needed to block NMDAr functions [3–6]. Also, AMPA receptors can be competitively inhibited by KYNA at millimolar concentrations, but in nanomolar to micromolar levels, KYNA induces their facilitation through allosteric modulation [7]. KYNA can also inhibit noncompetitively *α*7-nicotinic receptors (*α*7-nAChRs; IC_50_~7 *μ*M) which can bind to *α*-bungarotoxin being the most prevalent in the brain [5, 8, 9]. Under physiological conditions, it has been suggested that *α*7-nAChRs are the primary endogenous target of KYNA [10–12]. Due to KYNA can interact with NMDAr, *α*7-nAChRs and AMPAr[9,13,14]and since its levels secondarily affect the extracellular concentrations of glutamate, dopamine, acetylcholine and *γ*-aminobutyric acid (GABA) is considered as neuromodulator[10–19]. Importantly, all these receptors and neurotransmitters are critically involved in neurodevelopment, plasticity, cognition, behavior, and memory process among others [20].

On another hand, it has been shown that G-protein-coupled receptor (GPR35) is activated by KYNA [21]. The stimulation of this receptor is associated with neuronal excitability regulation and transmitter release, since GPR35 activation induces N-type calcium channel inhibition in rat sympathetic neurons [22, 23]. The KYNA effects on glutamate levels and the reduction of excitatory transmission can also be related with the ability of KYNA to activate GPR35 [23, 24]. In this regard, it has been proposed that the KYNA interaction with GPR35 reduces the release of proinflammatory cytokines in cell lines, which can be associated with the analgesic effects of KYNA in inflammatory models [25, 26]. Another target of KYNA is the aryl hydrocarbon receptor (AHR), which is considered a xenobiotic receptor [27], and its activation is associated with the suppression of cellular immune response favoring carcinogenesis and tumor outgrowth [25, 27]. Specifically, stimulation of AHR by KYNA enhances the expression of IL-6, fact by which KYNA was considered as a factor involved in the escape of tumors, via the IL-6-dependent pathway, for immune surveillance [27].

Finally, KYNA can also interact with reactive oxygen species (ROS) in chemical combinatory systems, and it can lead to decrease ROS production and lipid peroxidation induced by prooxidants, in rat brain homogenates. Importantly, this scavenger property of KYNA is independent of its effect on the NMDA and cholinergic receptors [28].

The relevance of KYNA in the brain has been experimentally shown both during development and adulthood. In this context, KYNA levels have been found higher in fetal brain [29–31] and decreased in the postnatal period and in adult age [31]. However, during adulthood, fluctuation of brain KYNA levels provokes a broad spectrum of behavioral and cognitive alterations [18, 32, 33], and when brain KYNA levels decreased, cognitive process improves in mice and rats [18, 34]. These evidences strongly suggest an important role of KYNA during neurodevelopment and adulthood.

**Figure 1:**
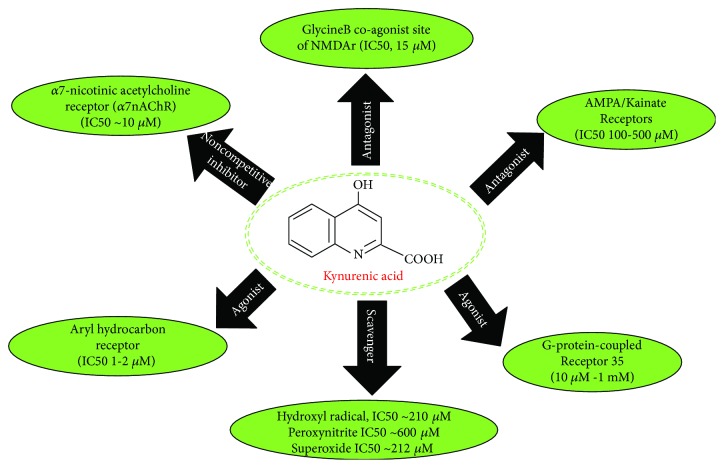
Targets of kynurenic acid (KYNA). AMPA: *α*-amino-3-hydroxy-5-methyl-4-isoxazolepropionic acid; *α*7nAChR: *α*7-nicotinic acetylcholine receptor; IC50: half maximal inhibitory concentration; NMDAr: N-methyl-D-aspartate receptor.

As was mentioned before, KYNA is an endogenous metabolite with multiple targets (Figure 1) that can lead to different effects depending on the environment conditions. Until now, the major production of KYNA has been attributed to kynurenine aminotransferases (KATs). Nevertheless, in events such as neurodevelopment, aging, some neurodegenerative diseases, and psychiatric disorders, the production of KYNA cannot be completely explained by the kynurenine aminotransferase activity alone but there are other common factors which could be involved in its production. In this review, we are focused on the alternative mechanisms by which KYNA can be produced since these could be extremely important under certain conditions.

## 2. Kynurenine Aminotransferase Canonical Way to Produce KYNA

The canonical route of KYNA formation is through the kynurenine pathway by kynurenine aminotransferases. These enzymes catalyze the irreversible transamination of kynurenine to produce KYNA. Until now, 4 isozymes of kynurenine aminotransferases have been described. All the isozymes are pyridoxal-5′-phosphate dependent and require an *α*-ketoacid molecule as a cosubstrate. KATs have a low affinity for their substrate (approximately 1 mM) so that the rate of KYNA formation is directly controlled by local kynurenine availability [35, 36]. Since the canonical pathway is the most studied, there are many reviews about these isozymes in the literature [37, 38]. Here, we provide a table with the principal biochemical characteristics of kynurenine aminotransferase isozymes (Table 1).

Recently, it has been reported that KATs can also take D-kynurenine (D-Kyn) as a substrate both in rat and in human tissues [39]. The de novo production of KYNA from D-Kyn in the rat prefrontal cortex was 30 times less potent than that from L-Kyn. The production induced by D-Kyn was inhibited just 30% by the KAT inhibitor AOAA, while the production induced by L-Kyn was almost abolished by the same treatment. Similar effects were observed in human homogenates—the production of KYNA from the enantiomer L-Kyn decreased around 98% in presence of the KAT inhibitor in the human brain and liver, while the KYNA production induced by D-Kyn drops at about 70% in presence of AOAA. Considering the low affinity for the substrate shown by the KATs and the evidence that the kynurenic acid produced by D-Kyn is not completely inhibited by the KAT inhibitor, it is feasible to suggest that there are alternative mechanisms by which KYNA can be produced and they could be relevant in physiological conditions as well as in pathological events.

## 3. D-Amino Acid Oxidase and D-Amino Acids in KYNA Production

During many years, L-amino acids have had more attention than D-enantiomers; however, recently, it has been shown that D-amino acids are present in animals and humans at high concentrations and fulfill specific biological functions, as was demonstrated with a pool of amino acids necessary for protein synthesis; after being enzymatically converted to L-amino acids, they could also act antagonistically to L-amino acids, deactivating their biological site [40, 41]. The presence of D-amino acids in mammals results from microorganisms or racemization of L-amino acids to their D-isomer, in food and other proteins which are pH, time, and temperature dependent [40]. Alterations in the concentrations of D-amino acids might occur in some disorders related to bacterial pathogens and immune activation [42].

Specifically for KYNA production, D-Trp and D-Kyn have been studied for many years. The first evidence showing that D-tryptophan (D-Trp) could be utilized for growth was reported by du Vigneaud and coworkers in 1932 [43]. But, it was Berg in 1953 [44] who demonstrated that D- and L-Trp can be equally effective to support growth in rats. After these findings, more studies on D-Trp were performed. It was found that in rat liver slices, D-Trp and D-Kyn were metabolized slower than L-Trp and L-Kyn, respectively. After incubation with D-Trp, small amounts of L-Kyn, D-Kyn, and KYNA were found [45]. In 1971, it was shown that after feeding or injecting rabbits with D-Trp or D-Kyn, they excreted kynurenic acid as well as indole pyruvic acid [46]. Also, in the normal human subject, it has been observed that, after ingestion of D-Trp, D-Kyn, indole pyruvic acid (IPA), and acetyl-Trp are excreted [47]. Later, it was showed that D-formylkynurenine was the intermediate during the conversion of D-Trp to D-Kyn, and the enzyme that catalyzed this reaction was inhibited by the presence of L-Trp [48]. *In vitro* experiments demonstrated that D-Kyn can be converted to KYNA in kidney preparations (slices and homogenates) and this conversion can be due to the presence of D-amino acid oxidase, since purified D-amino acid oxidase from *Trigonopsis variabilis* rapidly converts D-Kyn to KYNA [46]. These findings confirmed the previous results in which it was proposed that the mechanism by which D-Trp produced KYNA was independent of D-Trp racemization to L-Trp; however, this did not completely exclude the D- to L-Trp conversion possibility. Later, it was known that the conversion of L-Kyn to KYNA was catalyzed by *α*-ketoglutarate-dependent transaminase, and when L-Trp and L-Kyn were incubated in the presence of *α*-ketoglutarate-eliminating system, there was no KYNA production. On the contrary, when D-Trp or D-Kyn were incubated in the same conditions, KYNA production was found; however, when D-enantiomers were incubated in anaerobic conditions, KYNA was not detected, suggesting an alternative mechanism for KYNA production from D-enantiomers, which involved an oxidase and discarded the transaminase reaction [49].

Additional experiments showed that when rat liver homogenates were incubated with D-Trp (3 mg) under oxygen conditions, L-Trp, IPA, D-Kyn, KYNA, and anthranilic acid were produced. However, when the homogenates were incubated under nitrogen conditions, no metabolite was formed. To determine whether all inversion processes required oxygen, D-Trp was incubated with liver homogenates under oxygen conditions for 2 h; once IPA has been formed in this period of time, the atmosphere was changed to nitrogen and sodium azide was added to minimize kynurenine production; they could observe that even with the change of atmosphere, L-Trp continued to occur at expenses of IPA, which was formed under the oxidizing conditions. This means that the amination of IPA to L-Trp do not require aerobic conditions suggesting that the transamination occurs, since the reaction was stimulated by addition of glutamic acid and pyridoxal phosphate [50]. But the important point here was that not all D-Trp were inverted to L-Kyn; additionally, considerable amount of D-Kyn was also produced. When slices or homogenates of rat liver were incubated with D-Kyn, KYNA was produced, which was abolished by benzoate addition, indicating that D-amino acid oxidase (DAAO) was involved in the reaction, since benzoate is its inhibitor [50]. An *in vivo* experiment showed that the intraperitoneal (i.p.) administration of D-Trp or D-Kyn increased the levels of KYNA in rat plasma and this production was abolished by DAAO inhibitor, 5-methylpyrazole-3-carboxylic acid (summary in Table 2) [51, 52].

The first evidence that showed that D-Kyn can produce KYNA in rodents and the human brain was showed in 2009 [15, 53], and one year later, Pérez de la Cruz and coworkers found KYNA production from D-Kyn in different human brain regions, showing that in the human cerebellum, the production of KYNA is highest than in other regions. Furthermore, coincubation with benzoic acid inhibited KYNA production [54]. Moreover, microdialysis studies proved an increase in KYNA levels after intraperitoneal administration of D- or L-Trp (100 mg/kg) or direct infusion of D-Kyn in the prefrontal cortex. Interestingly, when the DAAO inhibitor was injected in combination with D-Trp or D-Kyn, the effect in KYNA levels was decreased [55]. Following this line, knowing that DAAO is abundant and has high activity in the cerebellum [56–58], microdialysis studies were designed in this region, showing that the infusion of 100 *μ*M of L-Kyn or D-Kyn produced 17.9 and 10.7 times more KYNA than the baseline, respectively, which was really surprising since a previous study demonstrated that KYNA production from D-Kyn needs 100 *μ*M of this enantiomer, while only 2 *μ*M of L-Kyn was necessary to produce almost the same amount of KYNA in rat cortex. Also, this experimental study showed the importance of DAAO in cerebellum KYNA production since *in vitro* experiments had shown that the production of KYNA from D-Kyn inhibited almost 30% by a KAT inhibitor, while it inhibited almost 70% by DAAO inhibitors [59].

Since these studies showed that KYNA can be produced in the brain from D-amino acids, new studies were focused on elucidating whether the other redox or neuroactive metabolites of the kynurenine pathway were also produced from D-enantiomers. In this context, Notarangelo and coworkers demonstrated that after i.p. D-Trp injection, the levels of L-Trp increased in the plasma, forebrain, and cerebellum, which confirmed that the conversion of D-Trp to L-Trp can take place in the brain and impact it. Then, they showed that D-Kyn increased both in the forebrain and in cerebellum and that at 30 min postinjection of D-Trp (30 mg/kg), KYNA levels were increased just in the cerebellum via DAAO activity, since the coadministration with a DAAO inhibitor decreased KYNA levels. The other branch of the pathway was also studied, and 3-HK and QUIN metabolites were increased in the forebrain after D-Trp injection; 3-HK increased 2-fold in the cerebellum, and any change in QUIN levels was not observed [42]. On the other hand, after i.p. D-Kyn injection, KYNA and 3-HK were found in the plasma, liver, forebrain, and cerebellum [60].

Since D-Trp and D-Kyn can be present in normal conditions by food intake or can be originated from microorganisms that inhabitate the digestive tract [61–64], it appears logical to suggest that D-enantiomers are, in part, responsible of KYNA, 3-HK, and QUIN levels in the brain. Knowing that kynurenine pathway metabolites have been associated with neurological disorders, it is also important to study the role of D-enantiomers since they can be responsible of the kynurenine level alterations in diseases, in which correlation with high DAAO activity or in those that are associated with previous infections is showed [65–67].

## 4. Indole-3-Pyruvic Acid as a KYNA Precursor

Indole pyruvic acid is a natural compound present in mammals and is the transamination product of tryptophan by the action of aromatic amino acid transaminase [68, 69]. The first studies that proposed that IPA could be a precursor of KYNA were conducted in the 1980s and demonstrated that IPA administration increased the brain content of 5-hydroxytryptamine (5-HT), 5-hydroxyindole-3-acetic acid (5-HIAA), and Trp [70, 71]. It was also demonstrated that Trp and IPA administration produced a dose-dependent increase of KYNA levels in the brain and others organs [71, 72]. Actually, the important point is that the same doses of IPA or Trp (100 mg/Kg) are able to produce almost the same concentrations of KYNA in rat brain (22 ± 2 and 23 ± 3 picomoles/g, resp.). At the same time, studies in parallel were carried out and rats were administrated with probenecid (inhibitor of KYNA's brain transport) and IPA, in order to clarify whether or not the raise in KYNA levels was due to an increased rate of synthesis or to a decreased rate of disposal. However, KYNA levels were significantly higher in animals treated with IPA + probenecid than in controls, suggesting not only that IPA indeed increases the rate of KYNA synthesis in rat brain but also that KYNA disposal occurs through a mechanism sensitive to probenecid. Until that time, it was known that the administration of IPA was able to increase KYNA levels but the mechanism was unknown. One of the hypotheses was that after administering IPA, Trp levels could be increased, which would generate a greater amount of KYNA by the canonical route. However, when ^3^H-IPA was administered and KYNA and Trp levels were monitored in the brain alkaline extracts, there were 2600 cpm/*μ*mol of KYNA and 380 cpm/*μ*mol of Trp found [72], suggesting that part of IPA could be converted to Trp and then it follows the canonical pathway to produce KYNA, but there was also another mechanism involved in KYNA production by IPA [71].

In this context, Politi and coworkers [73] showed that IPA could be transformed into KYNA in different rat organ homogenates, but in the absence of enzymatic systems and with oxygen in the incubation mixture. They incubated keto and enol forms of IPA in a free enzymatic system observing that the enol form produced more KYNA than the keto form (24 ± 5 ng and 6 ± 2 ng, resp.). Because the chemical transformation of IPA to KYNA needs a radical attack from reactive oxygen species, they also incubated in the same conditions keto and enol forms, but adding a free radical generator system (ascorbate/Fe/hydrogen peroxide). They observed that under these conditions, the enol form produced 251 ± 38 ng of KYNA while the keto form produced 12 ± 5 ng of KYNA. After these results, IPA scavenging properties were demonstrated through the inhibition of chemiluminescence and malondialdehyde formation; in both, the enol form was more efficient than the keto form, which is possibly due to the fact that the enol conformation contains two conjugated double bonds in the carbon frame [73].

**Figure 2:**
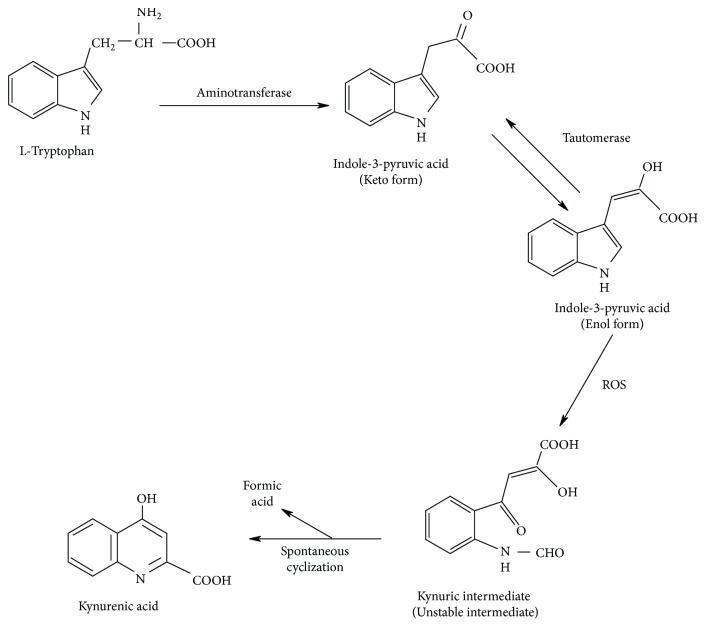
KYNA production from the interaction of indole pyruvic acid (IPA) with ROS (modified from Hardeland[74]).

In summary, tryptophan can be degraded by tryptophan 2-oxoglutarate aminotransferase, whose primary product is indole-3-pyruvic acid. IPA is either produced in keto or enol tautomer (Figure 2). The enolic form can easily interact with reactive oxygen species and undergoes pyrrole ring cleavage. The kynurenic product formed then spontaneously cyclizes to produce KYNA [74]. This process can be considered in mammals since it has been showed that IPA enol tautomer is rather stable in mammalian tissues and in plasma of mammals and humans treated with IPA, due to the presence of specific tautomerases in circulation, favoring the formation of KYNA in the presence of free radicals [75].

## 5. Myeloperoxidases Produce KYNA from L-Kyn

**Figure 3:**
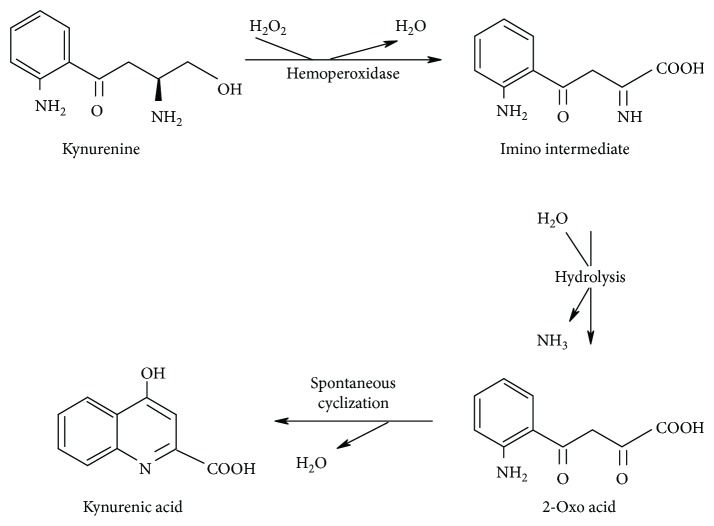
Hemoperoxidase participation on KYNA production from kynurenine (modified from Hardeland[74]).

The importance of peroxidases in KYNA production was evaluated after knowing that in homogenates of dinoflagellate *Lingulodinium polyedrum*, the KYNA production from L-Kyn was stimulated by oxidants [76]. After incubation of L-Kyn with H_2_O_2_ in the presence of peroxidases, KYNA production in a linear manner was observed. Taking in mind that hemoperoxidases, including horseradish peroxidase, have a broad substrate specificity for hydrogen donors, a mechanism by which these enzymes can produce KYNA from L-Kyn was proposed (Figure 3). Kynurenine can donate hydrogen forming an unstable imino acid, which is hydrolyzed to the respective 2-oxo acid and ammonia. Then, the 2-oxo acid formed spontaneously cyclizes and forms KYNA [74, 77]. This process can be considered in mammals, since hemoperoxidases may substantially favor the process in which H_2_O_2_ stimulates KYNA production.

## 6. Interaction between D-Kyn and L-Kyn with ROS Induces KYNA Production

As was mentioned before, L-Kyn can be converted to KYNA in the presence of H_2_O_2_, and this conversion is substantially enhanced by horseradish peroxidase. However, it is important to mention that this production was also observed in the absence of the enzyme. The reaction was monitored at different pHs, and the results showed that in acid pH (5.5), KYNA was not detectable; but when the pH of the medium was 7.4, 8, or 8.6, the KYNA production was increased at around 11- to 17.5-fold [77]. This evidence in the pH effect indicated that the major contribution in KYNA production from L-Kyn is due to H_2_O_2_ decomposition [78–81].

Later, cells of *Lingulodinium polyedrum* were incubated with kynurenine and KYNA levels were increased in the medium. This effect was highly light dependent. To clarify the relationship between photosynthetically generated oxygens during light and KYNA production from L-Kyn, Zsizsik and Hardeland evaluated the effect of two oxidant generators (carbonyl-cyanide-m-chlorophenylhydrazone (CCCP) and paraquat) and a photosynthesis inhibitor (dichlorophenyldimethylurea (DMCU)) in this paradigm. Incubation of L-Kyn in homogenates of *Lingulodinium polyedrum* exposed to light produced around 50–70 nmol KYNA/mg protein, and this production was stimulated in the presence of CCCP and paraquat (65% and 53%, resp.). However, KYNA production decreased around 42% in the presence of DMCU because this compound blocks the electron transport chain of photosystem II. This data suggested that oxidants (H_2_O_2_ and superoxide anions) stimulate KYNA production from L-Kyn [76].

Taking previous findings, Blanco Ayala and coworkers showed that the first evidence of the reaction between D-Kyn and L-Kyn with ROS produces KYNA in mammals [59]. By using chemical combinatorial assays, it was demonstrated that both D- and L-Kyn were able to produce KYNA through their interaction with hydroxyl radical and peroxynitrite, the effect with peroxynitrite being more pronounced. Then, cerebellum homogenates were used to evaluate the effect of coincubation of L- or D-Kyn with peroxynitrite. The production of KYNA from L-Kyn and D-Kyn in cerebellum homogenates was 18.1- and 9.8-fold higher, respectively, compared to the basal levels. When the homogenates were incubated with L- or D-Kyn plus peroxynitrite, the production increased by 2.6 and 2.8, respectively, compared with the incubation with the enantiomers alone. Next, through microdialysis experiments, it was demonstrated that the same effect occurs *in vivo.* Here, intracerebellar infusion of L- or D-Kyn produced KYNA level increments of 17.9 and 10.7 times, respectively, compared with baseline at 2 h postinfusion. In addition, basal levels of KYNA were increased in the cerebellum cortex (2.9 nM to 11.4 nM) after 30 min of peroxynitrite infusion, suggesting that the production of kynureninate is influenced by the oxidant environment. When the peroxynitrite was infused previously to both enantiomers, KYNA increased 4.1- and 3.2-fold compared with the animals infused just with L- or D-Kyn [59].

**Figure 4:**
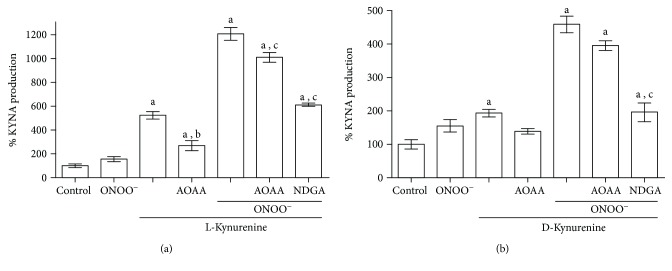
Effect of KAT inhibitor and a ROS scavenger on KYNA production from L-Kyn or D-Kyn in rat brain homogenates. AOAA and NDGA were used at 1 mM and 5 *μ*M, respectively. Homogenates were incubated with (a) L-Kyn or (b) D-Kyn (20 *μ*M and 10 *μ*M of ONOO^−^ in Krebs buffer) during 1 h at 37°C. Data are expressed as a percentage of endogenous tissue levels of KYNA and represent the mean ± SEM of 5 experiments per group. In both cases ^a^*P* < 0.05 versus control, ^b^*P* < 0.05 versus L-Kyn, and ^c^*P* < 0.05 versus L- or D-Kyn + ONOO^−^ (one-way ANOVA followed by Tukey's post hoc test). AOAA: aminooxyacetic acid; ONOO^−^: peroxynitrite; NDGA: nordihydroguaiaretic acid.

The importance of the redox environment was also observed in brain homogenates, which were incubated with 20 *μ*M of L- or D-Kyn and peroxynitrite (25 *μ*M) during 1 h at 37°C in Krebs buffer (Figure 4). Under these conditions, L-Kyn and D-Kyn increased KYNA levels 5- and 1.2-fold more, respectively, compared with those of the control. KYNA production from L-Kyn decreased by the use of AOAA, a KAT inhibitor, while KYNA production from D-Kyn in the presence of AOAA was not significantly altered. After coincubation with peroxynitrite, KYNA increases around 11- and 4-fold from L- and D-Kyn, respectively. The combination L-Kyn + ONOO^−^ + AOAA decreases just 20% KYNA production compared with L-Kyn + ONOO^−^, suggesting that KAT participation in KYNA production is minimal under these conditions. In the case of D-Kyn + ONOO^−^ + AOAA, it was not significantly different compared with D-Kyn and ONOO^−^. However, KYNA production from the enantiomers plus peroxynitrite was decreased around 50% when an antioxidant, NDGA, was used, suggesting that the KYNA production was favored by the oxidant environment.

These data are in accordance with previous evidence showing that L-Kyn and D-Kyn are good ROS scavengers and in this way can produce KYNA [82, 83]. All these findings suggest another pathway to produce KYNA which may have relevance in brain development and aging and in neurological diseases that show redox environment alteration.

## 7. Concluding Remarks

**Figure 5:**
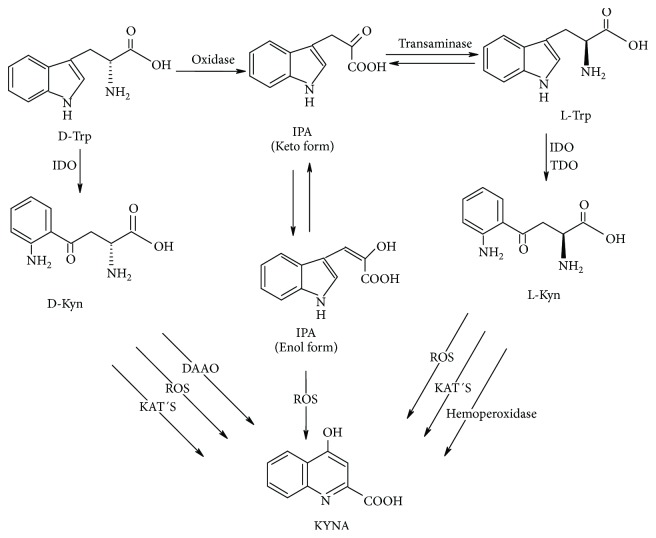
D-Trp can produce KYNA by two different ways: (1) D-Kyn formation by indoleamine dioxygenase (IDO) (since D-Kyn is a poor substrate of tryptophan dioxygenase (TDO). Once D-Kyn is formed, it can be taken as substrate by DAAO and KATs or to interact with ROS to produce KYNA and (2) the inversion of D-Trp to L-Trp, which occurs in two steps: the first one requires oxygen and it is suggested that DAAO can be the enzyme responsible to produce the intermediate IPA and the second step involves the reamination of IPA to yield L-Trp by means of a transaminase. L-Trp can follow the canonical pathway, that is, to produce L-Kyn, which is taken by the kynurenine aminotransferases (KATs) to produce KYNA. However, L-Kyn can also interact with ROS or with peroxidases and promote KYNA formation. The other important way to produce KYNA is through IPA, which in its enolic form can react with ROS producing KYNA.

Although the specific contributions of the alternative routes of KYNA production remain unclear, abundant evidence has shown that the increase of this metabolite is involved in many physiological and pathological processes, in which the redox environment is altered by the presence of free radicals, the decrease of antioxidant defense, and the activation of immune response and inflammatory mediators. All of these factors could be related with KYNA production as was mentioned throughout this review. The challenge for future research is to clarify the precise degree of involvement of these alternative routes (Figure 5), in processes such as neurodevelopment, aging, psychiatric disorders, and aging-related diseases, in which have been described as having high levels of KYNA; but also, it is known that there is high presence of free radicals and inflammatory cytokines. Some of these diseases are also related with previous infections and with DAAO activity alterations; all these factors promote the oxidant environment that could impact directly KYNA production. These new routes are a target of study and represent a new alternative to modulate KYNA levels in the processes in which they are involved.

## Figures and Tables

**Table 1:** Biochemical characteristic of KATs.

Enzyme	Cellular brain location	Characteristic	Optimum pH	Substrates with higher potency towards	Cosubstrate	Endogenous inhibitors	References
KAT-I/glutamine transaminase K/cysteine conjugate beta-lyase 1	Glia, astrocytes (nuclei and cytosol), and neurons (cytoplasmic)	(i) KAT I and KAT III share similar genomic structures (ii) KAT I immunoreactivity was found in ventral medulla, nucleus ambiguus, nucleus of the solitary tract, and intramedio lateral cell column of the spinal cord (iii) pKa 7.6 (iv) Enzyme partially purified: Km 875 *μ*mol/L, KYN concentrations ranging from 2 *μ*mol/L to 2 mmol/L (v) Relative percentage of brain activity: Mouse: 25.7 Rat: 15.5 Human: 9.8 (vi) Present high activity in the cerebellum	9.5–10	Glutamine	Pyruvate	Glutamine	[84–93]
				Phenylalanine		Tryptophan	
				Kynurenine (hKAT-I under physiological pH)		Phenylalanine	
						Indole-3-pyruvic acid	
						Cysteine	

KAT-II/*α*-aminoadipate aminotransferase	Astrocytes	(i) At physiologic KYN concentrations and pH, KAT II catalyzed around 75% of KYNA synthesis in most brain areas (ii) pKa 5.7 (iii) Enzyme partially purified: Km 660 *μ*mol/L, KYN concentrations ranging from 2 *μ*mol/L to 2 mmol/L (iv) Relative percentage of brain activity: Mouse: 12.3 Rat: 58.7 Human: 54.1	7.4	Kynurenine	*α*-Ketoglutarate	Aminoadipate	[85, 89, 90, 94, 95]
				Glutamate	Pyruvate	Asparagine	
				Aminoadipate		Glutamate	
				Methionine		Histidine	
						Cysteine	
						Lysine	
						3-Hydroxykynurenine phenylalanine	

KAT-III/cysteine conjugate beta-lyase 2		(i) mKAT III shows activity toward a number of amino acids (ii) mKAT III is more active than hKAT I under basic conditions (iii) Northern blot analysis showed a strong transcript in the liver, kidney, and heart and to a less extent in the brain and testis (iv) Has a higher isoelectric point than KAT I (v) 3-HK decreases mKAT III-catalyzed kynurenine transamination (vi) pKa 8.7	9-10	Glutamine	*α*-Ketobutyrate	Cysteine	[95–97]
				Histidine	Oxaloacetate	Glutamine	
				Methionine		Histidine	
				Phenylalanine		Methionine	
						Leucine	
						Phenylalanine	

KAT-IV/glutamic-oxaloacetic transaminase 2/mitochondrial aspartate aminotransferase	Mitochondria of astrocytes and neurons	(i) mKAT I, III, and IV showed high resistance to heat treatment (ii) pKa 6.9 (iii) Enzyme partially purified: Km 724 *μ*mol/L, KYN concentrations ranging from 2 *μ*mol/L to 2 mmol/L (iv) Relative percentage of brain activity: Mouse: 63.0 Rat: 25.8 Human: 36.1	8.5	Aspartate	*α*-Ketoglutarate	Aspartate	[94, 98]
				Glutamate		Glutamate	

**Table 2:** Enzymes involved in KYNA production from D- and L-enantiomers under different oxygen conditions.

	D-Trp → D-Kyn → KYNA	L-Trp → L-Kyn → KYNA	D-Trp → IPA	IPA → L-Trp
*α*-Ketoglutarate-eliminating system	✓	✘		
Aerobic conditions	✓	✓	✓	✓
Anaerobic conditions	✘	✓	✘	✓
Enzyme involved	DAAO	Transaminase	Oxidase	Transaminase
